# Th17 Cell Enhances CD8 T-Cell Cytotoxicity via IL-21 Production in Emphysema Mice

**DOI:** 10.1155/2012/898053

**Published:** 2012-12-25

**Authors:** Min-Chao Duan, Ying Huang, Xiao-Ning Zhong, Hai-Juan Tang

**Affiliations:** Department of Respiratory Medicine, The First Affiliated Hospital of Guangxi Medical University, Nanning, Guangxi 530021, China

## Abstract

Emphysema is a T-cell mediated autoimmune disease caused predominantly by cigarette smoking. Th17 cells and related cytokines may contribute to this disorder. However, the possible implication of Th17 cells in regulating inflammatory response in emphysema remains to be elucidated. In the current study, we tested the protein levels of IL-17 and IL-21 in peripheral blood and lung tissues from cigarette-smoke- (CS-) exposed mice and air-exposed mice, analyzed the frequencies of CD4^+^IL-17^+^(Th17) cells, IL-21^+^Th17 cells, and CD8^+^IL-21R^+^ T cells in peripheral blood and lung tissues of mice, and their relationship with emphysematous lesions, and explored the impact of IL-21 on cytotoxic CD8^+^ T cells function *in vitro.* It was found that the frequencies of Th17, IL-21^+^Th17, and CD8^+^IL-21R^+^ T cells and the levels of IL-17 and IL-21 of CS-exposed mice were much higher than those of the air-exposed mice and correlated with emphysematous lesions. Additionally, the number of IL-21^+^Th17 cells positively correlated with the number of CD8^+^IL-21R^+^ T cells. The *in vitro* experiments showed that IL-21 significantly augmented the secretion of perforin and granzyme B in CD8^+^ T cells from CS-exposed mice. These data indirectly provide evidence that Th17 cells could be involved in the control of the local and system inflammatory response in emphysema by regulating CD8^+^ cytotoxic T-cell function.

## 1. Introduction

Chronic obstructive pulmonary disease (COPD) is a leading cause of disability and death worldwide [1–3]. It is predominantly caused by smoking and is characterized by poorly reversible airflow limitation. Pulmonary emphysema is a major component of COPD. Although COPD is more and more common, the molecular and cellular mechanisms that are responsible for the development of COPD are not well understood. Early studies have shown that COPD is marked by the accumulation of both CD4^+^ and CD8^+^ T cells in the airways and lung parenchyma, with CD8^+^ T cells predominating [4]. Recent findings suggest that COPD is an autoimmune disease characterized by an association of antielastin antibody and Th1 response [5, 6]. Th1 cells contribute principally, but not exclusively, to the pathogenesis of COPD.

IL-17 (also known as IL-17A) was first cloned in 1993 and identified as cytotoxic T lymphocyte-associated antigen (CTLA)-8 [7]. IL-17F was later discovered and closely related with IL-17A. They are all expressed in activated CD4^+^ memory T cells [8]. Increasing evidence has indicated that IL-17 is involved in inflammatory disorders of the lungs [9, 10]. IL-17 may play an important role in the pathogenesis of COPD because of its ability to induce neutrophilic airway inflammation by stimulation of neutrophil chemotaxis and mucin gene expression in bronchial epithelial cells [11, 12]. Overexpression of IL-17 in lungs of transgenic mice may induce lung inflammation with a COPD-like phenotype [13]. Th17 cells are the newly described subset of CD4^+^ T cells and have significant role in the progression of several T cells driven by autoimmune diseases, such as rheumatoid arthritis and multiple sclerosis, which were previously thought to be exclusively mediated by Th1 cells [14, 15]. More recently, Th17 cells have been found in COPD and emphysema [16, 17]. However, the role of Th17 cells in regulating inflammatory response in emphysema remains to be demonstrated.

IL-21 is a pleiotropic cytokine of the *γ*-chain family, which engages the common cytokine receptor *γ*-chain expressed on cells of both lymphoid and myeloid lineages. This cytokine was originally thought to be restricted to CD4^+^ T cells (Th1 and Th2 cells) and NKT cells, but it is now clear that IL-21 is also produced by Th17 cells [18, 19]. IL-21 can serve to recruit Th17 cells into the inflamed tissue, and also deliver intracellular signal through IL-21R and influence T-cell activation and differentiation [20]. Recently, Zeng et al. [21] demonstrated that *in vitro* exposure to IL-21 can lead to the generation of CD8^+^ T cell in increased numbers and with enhanced function. These data suggest that Th17 cells may play role in regulating CD8^+^ cytotoxic T-cell function via IL-21/IL-21R. 

In the present study, we hypothesized that Th17 cells and IL-21 are involved in the local and system inflammatory response using a murine model of emphysema induced by smoking. We tested IL-17 and IL-21 protein levels in both peripheral blood and lung tissues of cigarette-smoke- (CS-) exposed mice and air-exposed mice, and analyzed the expression of Th17, IL-21^+^Th17, and CD8^+^IL-21R^+^ T cells in peripheral blood and lung tissues of mice, and their relationship with emphysematous lesions. Finally, we assessed the possible modulating effect of recombinant IL-21 (rIL-21) on CD8^+^ T cells *in vitro*.

## 2. Materials and Methods

### 2.1. Animals and Cigarette Smoke Exposure Protocol

Specific pathogen-free inbred male BALB/c mice (8 weeks of age, 20–25 g body weight) were purchased from the Guangxi Medical University Laboratory Animal Center (Nanning, China). All animal procedures were reviewed and approved by the Laboratory Animal Ethics Committee of Guangxi Medical University. All mice were housed in sterilized cages and maintained on a 12 : 12 h light-dark cycle and received sterilized food and water ad libitum.

Mice (*n* = 10) were exposed whole-body to CS, as described previously [22]. Briefly, groups of ten mice (CS-exposed mice) were exposed to five cigarettes (Nanning Jiatianxia unfiltered cigarettes: 12 mg of tar and 0.9 mg of nicotine), 4 times a day with 30 min smoke-free intervals in a closed 0.75 m^3^ room, 5 days a week for 24 weeks. Mice tolerated cigarette smoke exposure without evidence of toxicity (carboxyhemoglobin levels ~10% and no weight loss). An optimal smoke : air ratio of 1 : 6 was obtained. The control groups (air-exposed mice) were exposed to 24 weeks air. The serum carboxyhemoglobin of CS-exposed mice was 8.4 ± 1.2% versus 1.0 ± 0.3% in air-exposed mice (*n* = 10), which is similar to carboxyhemoglobin blood concentrations of human smokers [23].

### 2.2. Tissue Processing

24 hours after the last air or smoke exposure, the mice were sacrificed with sodium pentobarbital anesthesia. Blood samples were collected via retroorbital bleeding and were divided into 2 parts. Approximately 200 *μ*L of peripheral blood was obtained for flow cytometric analysis, and approximately 1500 *μ*L of peripheral blood was separated serum for enzyme-linked immunosorbent assay analysis (ELISA). The left lungs were used for histology. One part of the right lungs (30 to 50 mg) was homogenized for ELISA, and remanent parts were used for the preparation of single-cell suspensions. Spleens were harvested aseptically and minced for culture.

### 2.3. Morphometry

The left lungs were inflated by instilling 10% formalin at a constant pressure of 25 cm H_2_O (for 10 min) and then ligated and removed. Inflated lungs were fixed for 24 h before embedding in paraffin. After paraffin embedding, 5 *μ*m sections were cut and stained with hematoxylin and eosin for histological analysis. For each animal, 10 fields at a magnification of 100x were captured randomly from the 4 different zones of the left lung. We determined enlargement of alveolar spaces by quantifying the mean linear intercept (Lm) and destruction of alveolar walls by measuring the destructive index (DI) in CS- and air-exposed mice, as described previously [24, 25]. Two investigators independently measured Lm and DI in a blinded manner.

### 2.4. Preparation of Lung Single-Cell Suspensions

Lung single-cell suspensions were prepared from part of the right lung, as detailed previously [26]. Briefly, the lung was thoroughly minced, digested, passed through a 70 *μ*m cell strainer, washed and centrifuged twice with cold PBS at 1200 rpm for 10 min at 4°C, and resuspended in PBS. The mononuclear cells were isolated from the lung single-cell suspension by Ficoll-Hypaque gradient centrifugation (Pharmacia, Uppsala, Sweden), washed and centrifuged twice with cold PBS at 1200 rpm for 10 min at 4°C, and kept on ice until labelling.

### 2.5. Lymphocyte Preparation

Erythrocytes were lysed with RBC lysis buffer (Sigma-Aldrich) for 10 minutes at room temperature and the remaining cells were washed twice with cold PBS and centrifuged at 1200 rpm for 10 minutes. Fresh peripheral-blood mononuclear cells (PBMCs) were used for intracellular cytokine staining within 1 h.

After mincing spleens, the cell suspensions were pipetted rapidly with a sterile Pasteur pipette into 3 mL of RPMI 1640 (Gibco, USA), filtered through nylon mesh to eliminate debris, and centrifuged at 1000 rpm for 5 min. The cell pellets of spleens were resuspended in PBS, and the lymphocyte fractions were isolated by Ficoll-Plaque (Solarbio Science & Technology, China) gradient centrifugation. Lymphocytes were maintained in a 24-well flat-bottom tissue culture plate with RPMI 1640 supplemented with 10% fetal calf serum (Gibco, USA) at 37°C in a humidified atmosphere with 5% CO_2_.

### 2.6. Immunofluorescence Labeling and Flow Cytometry

The expression markers on T cells were determined by flow cytometry after surface staining or intracellular staining using phycoerythrin cyanine-5-conjugated anti-mouse CD4 (PE-Cy5-CD4), fluorescein isothiocyanate-conjugated anti-mouse CD8 (FITC-CD8), phycoerythrin-conjugated anti-mouse IL-17 (PE-IL-17), phycoerythrin-conjugated anti-mouse IL-21R (PE-IL-21R), and Alexa Fluor 647-conjugated anti-mouse IL-21 (Alexa Fluor 647-IL-21). These mice Abs were purchased from BD Biosciences or eBioscience (San Diego, CA). Briefly, PBMCs were stimulated with phorbol myristate acetate (PMA, 25 ng/mL, Sigma-Aldrich, USA) and ionomycin (10 *μ*g/mL, Sigma-Aldrich, USA) in the presence of GolgiStop (BD Biosciences) for 5 h. The cells were washed and then fixed/permeabilized in the eBioscience fixation/permeabilization and permeabilization buffers according to the manufacturer's protocol [27], stained with fluorescent antibodies against CD4, CD8, IL-21R, IL-17, and IL-21. Flow cytometry was performed on a BD FACSCalibur flowcytometer and analyzed by using FCS ExpressV4 software.

### 2.7. Spleen CD8^+^ T Cell Cultures and rIL-21 Stimulation

Bulk CD8^+^ T cells from spleen were positively selected using paramagnetic microbeads conjugated to anti-mouse CD8 (Ly-2) monoclonal antibody according to the manufacturer's instructions (MACS, Miltenyi Biotec). The purity of CD8^+^ T cells thus obtained was approximately 95%.

Purified CD8^+^ T cells were cultured at 1 × 10^6^ cells/mL in RPMI 1640 medium containing 10% FBS, 100 U/mL penicillin, 100 g/mL streptomycin, 2 mM L-glutamine, and 50 M mercaptoethanol (RPMI 1640 complete medium 2 mL) in 96-well plates (200 *μ*L) at 37°C, 5% CO_2_, and 100% humidity with phytohemagglutinin (PHA, 10 ng/mL) and treated with or without 50 ng/mL of mrIL-21 for 3 days. A cytokine concentration of 50 ng/mL was chosen based on initial dose-response experiments with 10 to 100 ng/mL concentrations and published literature to achieve maximal effect on CD8 T-cell cytotoxic [21, 28]. The cells were washed once and restimulated with PMA/ionomycin in the presence of GolgiStop (BD Biosciences) for 5 h and then fixed/permeabilized with eBioscience fixation/permeabilization according to the manufacturer's protocol and stained with antibodies specific for intracellular perforin (PE-perforin) and granzyme B (APC-granzyme B).

### 2.8. Cytokine Measurement

The concentrations of IL-17 and IL-21 in the peripheral blood and the lungs, as well as perforin and granzyme B in culture supernatants, were measured by ELISA kits according to the manufacturer's protocols (R&D Systems, Minneapolis, MN). All samples were assayed in duplicate.

### 2.9. Statistical Analysis

All data were described as the mean ± SD. Independent-samples *t*-test and Pearson correlation were used for statistical analysis. Statistical analysis was performed by using SPSS statistical software version 16 (SPSS Inc., Chicago, IL), and *P* values <0.05 were considered as significant.

## 3. Results

### 3.1. Histological and Lung Morphometric Studies

**Figure 1:**
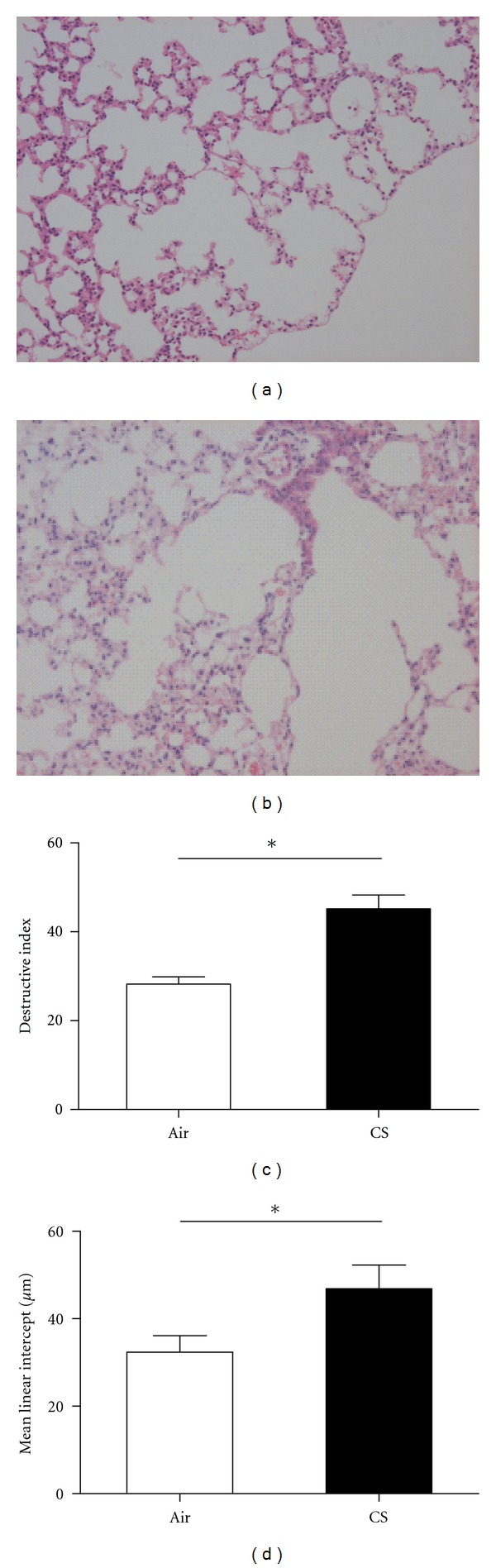
Photomicrographs of haematoxylin and eosin-stained lung tissue of air- and CS-exposed mice at 24 weeks (magnification, ×100). Smoke exposure clearly induced alveolar wall destruction and airspace enlargement in mice. (a) Air-exposed mice, (b) CS-exposed mice. Quantification of pulmonary emphysema. Morphometry of the lungs after chronic (24 weeks) air or CS exposure: (c) DI and (d) Lm values of mice. Results are expressed as means ± SD. *n* = 10 animals/group; *P < 0.001.

Emphysema is a structural disorder characterized by destruction of the alveolar walls and enlargement of the alveolar spaces. Histologically, the lungs sections from the air-exposed mice showed normal alveolar structure and exhibited normal size airspaces with thin septa (Figure 1(a)). In contrast, the lungs sections from the CS-exposed mice showed an increased air space enlargement and destruction. Some airspaces seemed irregular in size, and septa were thin (Figure 1(b)). The DI was higher in CS-exposed mice (45.16 ± 3.13) compared with air-exposed animals (28.86 ± 2.07, *P* < 0.001; Figure 1(c)); exposure to cigarette smoke significantly induced airspace enlargement. The Lm was also higher in CS-exposed mice (46.87 ± 7.16 *μ*m) compared with air-exposed animals (32.60 ± 3.21 *μ*m, *P* < 0.001; Figure 1(d)).

### 3.2. IL-17 and IL-21 Protein Levels Were Significantly Elevated in CS-Exposed Mice

**Figure 2:**
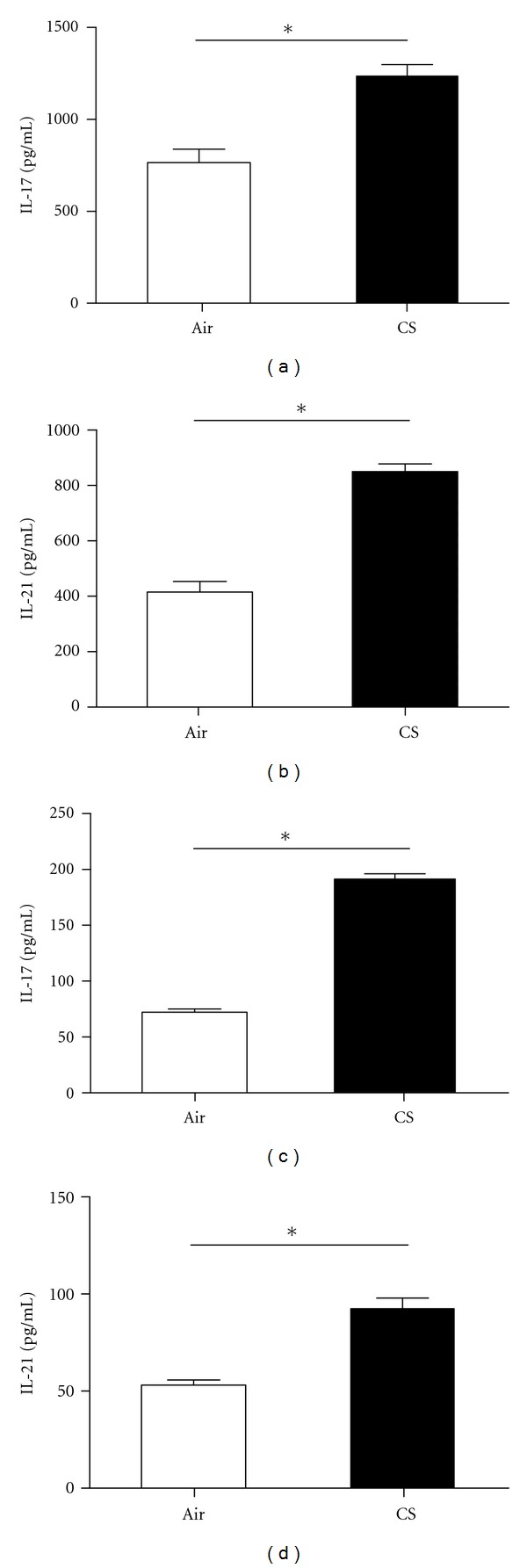
Protein levels of Interleukin- (IL-) 17 and Interleukin-21 (IL-21) in lungs and peripheral blood of air- and CS-exposed mice at 24 weeks by using ELISA. (a) Protein levels of IL-17 in lungs, (b) protein levels of IL-21 in lungs, (c) protein levels of IL-17 in peripheral blood, and (d) protein levels of IL-21 in peripheral blood. Results are expressed as pg/mL (mean ± SD). *n* = 10 animals/group; *P < 0.001.

In the present study, using ELISA we noted that the lung levels of IL-17 and IL-21 were significantly increased in CS-exposed mice compared to air-exposed mice (*P* < 0.001, Figures 2(a) and 2(b)). Similarly, we also noted that the levels of IL-17 and IL-21 in the peripheral blood of CS-exposed mice were significantly higher than those of air-exposed mice (*P* < 0.001, Figures 2(c) and 2(d)).

**Figure 3:**
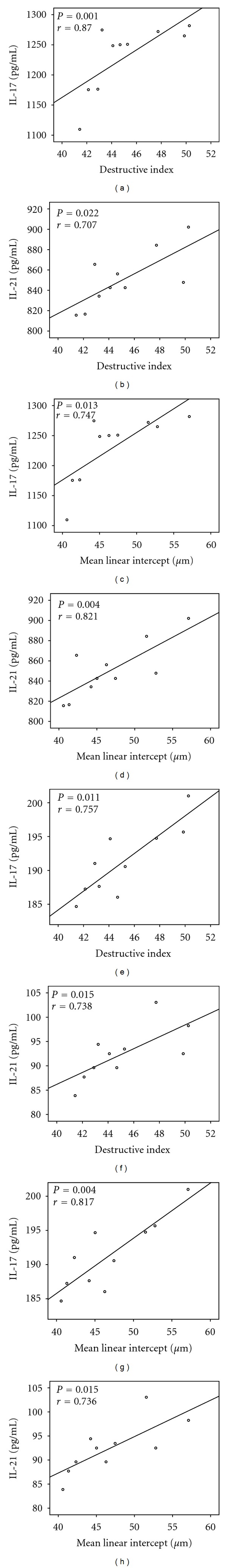
Correlations between (a) the protein levels of IL-17 in lungs and DI, (b) the protein levels of IL-21 in lungs and DI, (c) the protein levels of IL-17 in lungs and Lm, (d) the protein levels of IL-21 in lungs and Lm, (e) the protein levels of IL-17 in peripheral blood and DI, (f) the protein levels of IL-21 in peripheral blood and DI, (g) the protein levels of IL-17 in peripheral blood and Lm, and (h) the protein levels of IL-21 in peripheral blood and Lm. Data were determined by Pearson's rank correlation coefficients.

To further confirm our results, we studied the relation between the levels of IL-17 and IL-21 and emphysematous lesions as measured by DI and Lm in CS-exposed mice. The lung levels of IL-17 and IL-21 were positively correlated with DI (*r* = 0.87, *P* = 0.001 and *r* = 0.707, *P* = 0.022, resp., Figures 3(a) and 3(b)) and with Lm (*r* = 0.747, *P* = 0.013 and *r* = 0.821, *P* = 0.004, resp., Figures 3(c) and 3(d)). Similarly, the peripheral blood levels of IL-17 and IL-21 were positively correlated with DI (*r* = 0.757, *P* = 0.011 and *r* = 0.738, *P* = 0.015, resp., Figures 3(e) and 3(f)) and with Lm (*r* = 0.817, *P* = 0.004 and *r* = 0.736, *P* = 0.015, resp., Figures 3(g) and 3(h)).

### 3.3. The Frequencies of Th17, IL-21^+^Th17, and CD8^+^IL-21R^+^ T Cells Were Increased in CS-Exposed Mice

**Figure 4:**
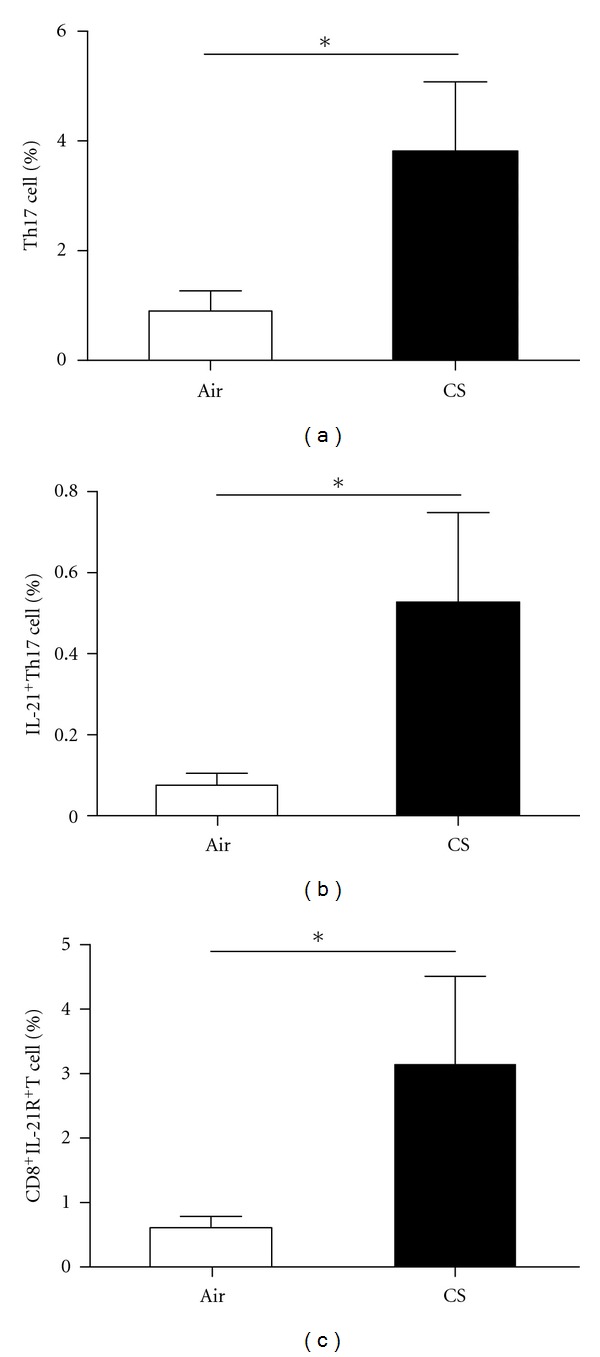
The frequency of CD4^+^IL-17^+^(Th17), cells, IL-21^+^Th17 cells and CD8^+^IL-21R^+^ T cells in peripheral blood was measured by using flow cytometry. The frequency (%) of CD4^+^IL-17^+^(Th17) cells (a), IL-21^+^Th17 cells (b), and CD8^+^IL-21R^+^ T cells (c) increased in peripheral blood of CS-exposed mice compared with air-exposed mice. Results are expressed as % (mean ± SD). *n* = 10 animals/group; *P < 0.05.

**Figure 5:**
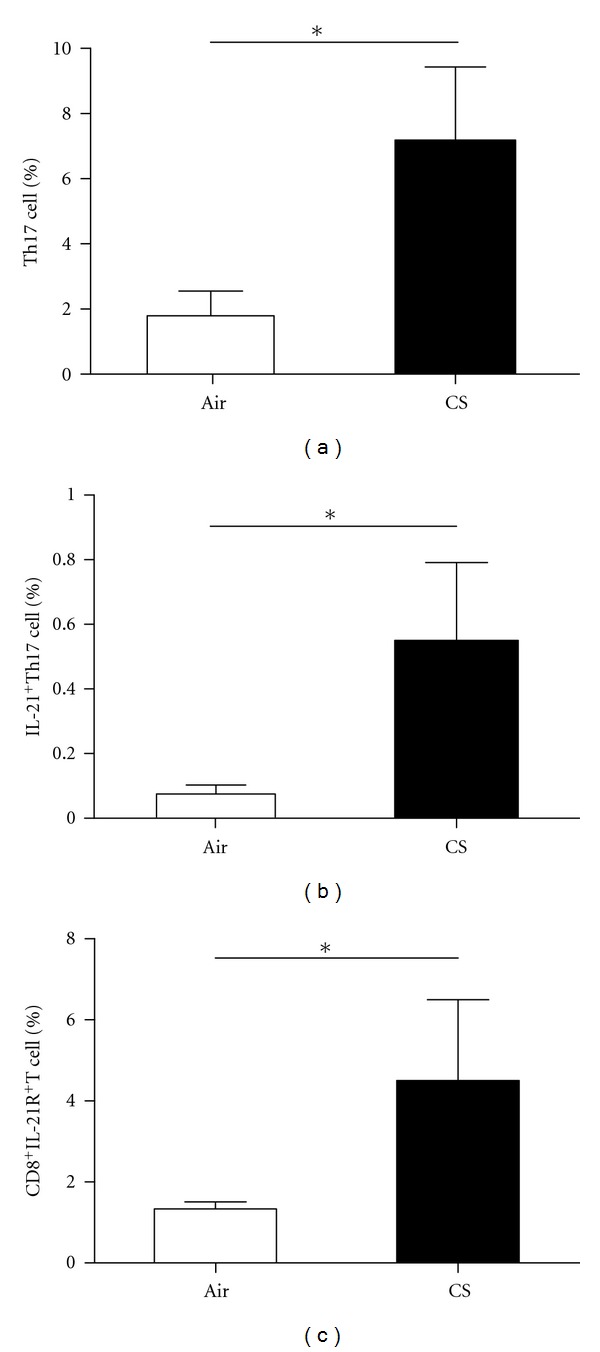
The frequencies of CD4^+^IL-17^+^(Th17) cells, IL-21^+^Th17 cells, and CD8^+^IL-21R^+^ T cells in lungs were measured by using flow cytometry. The frequencies (%) of CD4^+^IL-17^+^(Th17) cells (a), IL-21^+^Th17 cells (b), and CD8^+^IL-21R^+^ T cells (c) increased in lungs of CS-exposed mice compared with air-exposed mice. Results are expressed as % (mean ± SD). *n* = 10 animals/group; *P < 0.05.

We analyzed the frequencies of Th17, IL-21^+^Th17, and CD8^+^IL-21R^+^ T cells in the peripheral blood and the lungs by using flow cytometry. The frequencies of Th17 and IL-21^+^Th17 cells in the peripheral blood of CS-exposed mice were significantly increased compared to air-exposed littermates (*P* < 0.05, Figures 4(a) and 4(b)). Also after chronic CS-exposure for 6 months, a significant increase in CD8^+^IL-21R^+^ T cells was observed in the peripheral blood from CS-exposed mice compared with those in air-exposed littermates (*P* < 0.001, Figure 4(c)). In addition, the frequencies of Th17, IL-21^+^Th17, and CD8^+^IL-21R^+^ T cells in the lungs of CS-exposed mice were significantly higher than those of the controls (*P* < 0.05, Figures 5(a), 5(b), and 5(c)).

### 3.4. Correlation between Frequencies of Th17, IL-21^+^Th17, and CD8^+^IL-21R^+^ T Cells and Emphysematous Lesions in CS-Exposed Mice

**Figure 6:**
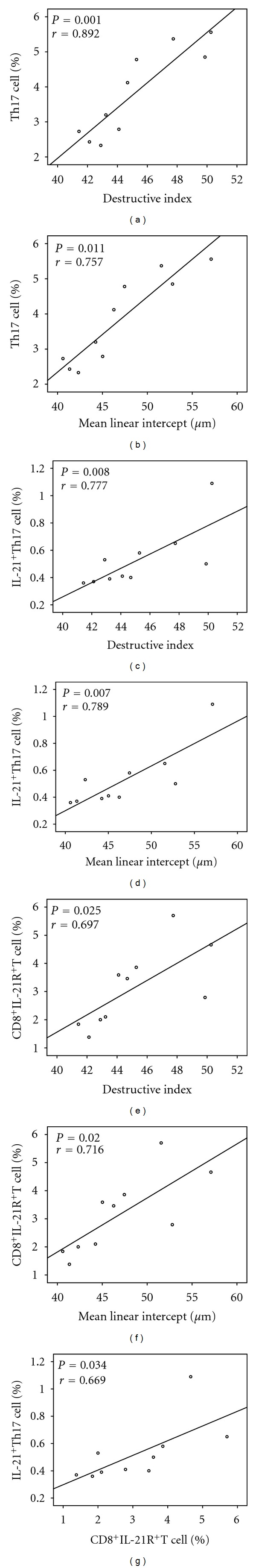
Correlations between (a) the frequency of CD4^+^IL-17^+^Th17 cells in peripheral blood and DI, (b) the frequency of CD4^+^IL-17^+^Th17 cells in peripheral blood and Lm, (c) the frequency of IL-21^+^Th17 cells in peripheral blood and DI, (d) the frequency of IL-21^+^Th17 cells in peripheral blood and Lm, (e) the frequency of CD8^+^IL-21R^+^ T cells in peripheral blood and DI, (f) the frequency of CD8^+^IL-21R^+^ T cells in peripheral blood and Lm, and (g) the frequency of CD8^+^IL-21R^+^ T cells and the frequency of IL-21^+^Th17 cells in peripheral blood. Data were determined by Pearson's rank correlation coefficients.

The frequencies of peripheral blood Th17, IL-21^+^Th17, and CD8^+^IL-21R^+^ T cells were positively correlated with DI (*r* = 0.892, *P* = 0.001; *r* = 0.777, *P* = 0.008 and *r* = 0.697, *P* = 0.025, resp.) (Figures 6(a), 6(c), and 6(e)) and with Lm (*r* = 0.757, *P* = 0.011; *r* = 0.789, *P* = 0.007 and *r* = 0.716, *P* = 0.020, resp.) (Figures 6(b), 6(d), and 6(f)) in CS-exposed mice. In addition, IL-21^+^Th17 cells were correlated positively with CD8^+^IL-21R^+^ T cells (*r* = 0.648,  *P* = 0.005) (Figure 6(g)).

**Figure 7:**
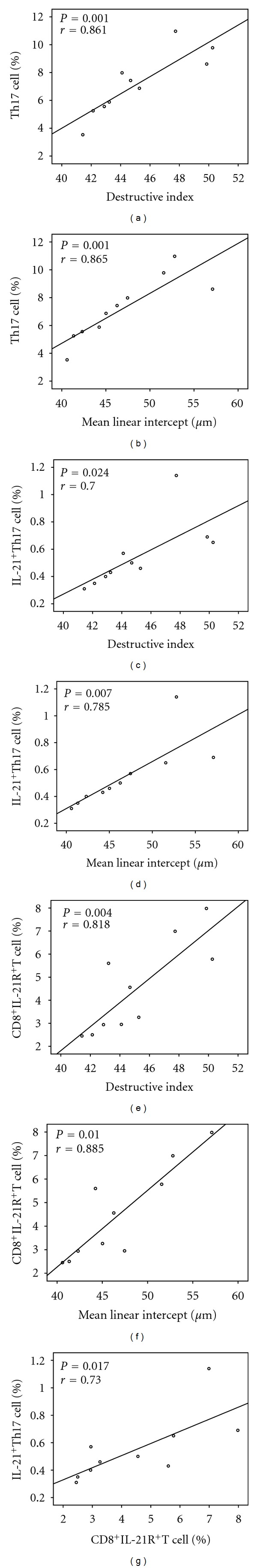
Correlations between (a) the frequency of CD4^+^IL-17^+^Th17 cells in lungs and DI, (b) the frequency of CD4^+^IL-17^+^Th17 cells in lungs and Lm, (c) the frequency of IL-21^+^Th17 cells in lungs and DI, (d) the frequency of IL-21^+^Th17 cells in lungs and Lm, (e) the frequency of CD8^+^IL-21R^+^ T cells in lungs and DI, (f) the frequency of CD8^+^IL-21R^+^ T cells in lungs and Lm, and (g) the frequency of CD8^+^IL-21R^+^ T cells and the frequency of IL-21^+^ Th17 cells in lungs. Data were determined by Pearson's rank correlation coeficients.

The frequencies of lung Th17, IL-21^+^Th17, and CD8^+^IL-21R^+^ T cells were significantly and positively correlated with DI (*r* = 0.861, *P* = 0.001; *r* = 0.700,  *P* = 0.024 and *r* = 0.818, *P* = 0.004, resp.) (Figures 7(a), 7(c), and 7(e)) and with Lm (*r* = 0.865, *P* = 0.001; *r* = 0.785, *P* = 0.007 and *r* = 0.885, *P* = 0.001, resp.) (Figures 7(b), 7(d), and 7(f)) in CS-exposed mice. In addition, IL-21^+^Th17 cells were correlated positively with CD8^+^IL-21R^+^ T cells (*r* = 0.73, *P* = 0.017) (Figure 7(g)). Our findings indicated that Th17 cells might be able to regulate CD8^+^T cells via IL-21/IL-21R system.

### 3.5. IL-21 Upregulates Perforin and Granzyme B Expression in CD8^+^ T Cells

**Figure 8:**
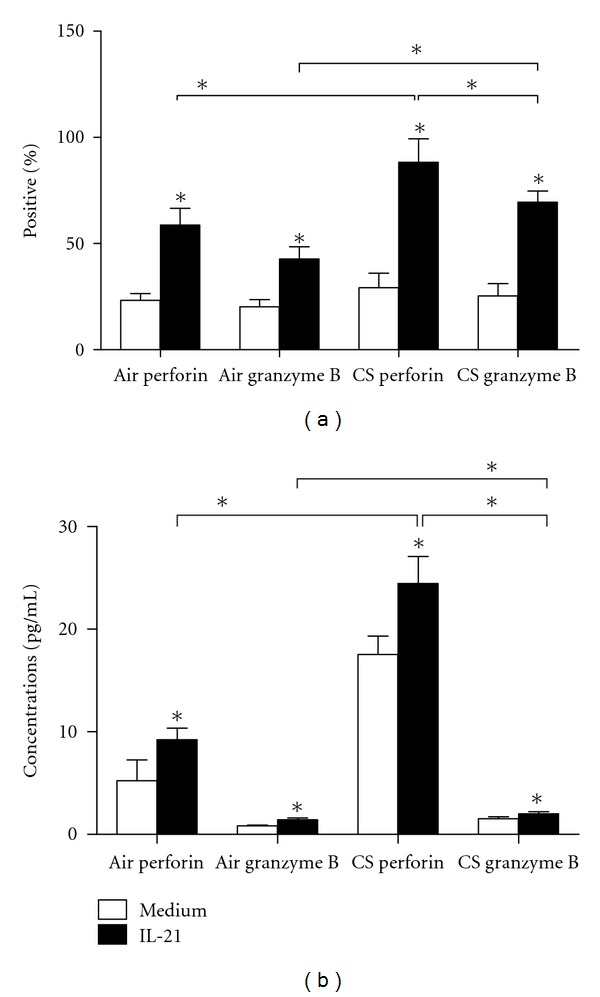
IL-21 upregulates perforin and granzyme B expression and protein levels in CD8^+^ T cells. The purified CD8^+^ T cells were cultured in the presence of PHA (10 ng/mL) and rIL-21(50 ng/mL) or PHA (10 ng/mL) alone for 3 days and analyzed for (a) perforin and granzyme B expression in CD8^+^ T cells of air-exposed and CS-exposed mice (*n* = 10). (b) Perforin and granzyme B concentrations in CD8^+^ T cells of air-exposed and CS-exposed mice (*n* = 10). Significance was determined by independent-samples *t*-test.

To evaluate the contribution of IL-21 to the function of CD8^+^ T cells in CS-exposed mice, we next cultured CD8^+^ T cells *in vitro* with rIL-21 to analyze cytokine production. We isolated CD8^+^ T cells by MACS from CS-exposed mice and from air-exposed mice. The purified CD8^+^ T cells were cultured in the presence of PHA (10 ng/mL) and rIL-21 (50 ng/mL) or PHA (10 ng/mL) alone for 3 days. Results showed that administration of rIL-21 and PHA significantly upregulated the expression of perforin and granzyme B in CD8^+^ T cells; this effect was greater in CS-exposed mice than in air-exposed mice (Figure 8(a)). Notably, there were more perforin+ cells (88.29 ± 11.03%) than granzyme B+ cells (69.47 ± 5.31%, *P* < 0.01) in CD8^+^ T cells of CS-exposed mice (Figure 8(a)). In parallel, the protein levels of perforin and granzyme B in the culture supernatants significantly increased following 3 days of culture with IL-21 and PHA; this effect was also greater in CS-exposed mice than in air-exposed mice (Figure 8(b)). The protein levels of perforin in the culture supernatants of CS-exposed mice (24.47 ± 2.61 ng/L) were significantly increased more than the levels of granzyme B (1.92 ± 0.21 ng/L, *P* < 0.001) (Figure 8(b)). These data suggest that IL-21R^+^CD8^+^T cells have the capacity to secrete perforin and granzymes and further support the potential relevance of Th17 cells in emphysema.

## 4. Discussion

Emphysema is considered a T-cell-mediated autoimmune disease, but its etiology and pathology have not been elucidated. In this study, we employed a murine model of cigarette smoke-induced lung emphysema to investigate the capacity of Th17 cells to participate in emphysema pathogenesis. We found that the numbers of Th17, IL-21^+^Th17, and CD8^+^IL-21R^+^ T cells and the levels of IL-17 and IL-21 in the peripheral blood and lungs of CS-exposed mice were much higher than those of air-exposed mice and correlated with emphysematous lesions. Additionally, the number of IL-21^+^Th17 cells positively correlated with the number of CD8^+^IL-21R^+^ T cells. Furthermore, IL-21 significantly augmented the secretion of perforin and granzyme B in CD8^+^ T cells from CS-exposed mice *in vitro*. These data indirectly demonstrate that IL-21 produced by Th17 cells can act on CD8^+^ T cells to promote cytotoxic function.

CD4^+^ T cells are known as important key cells in immunoregulation, whereas CD8^+^ T cells have cytotoxic function in COPD [29]. Classically, naive CD4^+^ T cells have been thought to differentiate into two main lineages, Th1 and Th2 cells on the basis of their cytokines secretion and immune regulatory function [30]. Regulatory T cells represent only a small subset of CD4^+^ T cells in the peripheral circulation and are responsible for the balance of immune responses, which is essential for health [31]. Th17 cell changes the classical Th1/Th2 paradigm of Th cell differentiation [32]. Early studies have suggested that infiltrating CD4^+^ T cells in COPD exhibit a Th1 phenotype [29]. Our previous study showed that decreased regulatory T cells were found in lungs of emphysema group [33]. More recently, we have reported that increased Th17 cells could be found in lungs of smoke-exposed mice, and these Th17 cells might be due to Th17 differentiation stimulated by lung proinflammatory cytokines and to recruitment of Th17 cells via CCR6/CCL20 [34]. In the current study, we also demonstrated that increased Th17 cells were present in peripheral blood and lungs of CS-exposed mice. More important, the increased frequency of Th17 cells positively correlated with emphysematous lesions. These findings are in agreement with the *in vivo* data of Shan et al. [17], who demonstrated that Th17 cells were present in lungs from patients with emphysema, and Harrison et al. [35] also found that Th17 cells presented in the BALF from smoke-exposed mice. Thus, these studies indicated that Th17 cells may have a relevant role in the local and system inflammatory process of COPD.

Th17 cells secrete not only IL-17A but also IL-17F, IL-21, and IL-22, these cytokines most likely induce tissue inflammation [36]. Recent studies have been showed that IL-17A and IL-17F could stimulate chemokine production and promote neutrophil and macrophage recruitment to the lung [37, 38]. But additional roles for this and other Th17-derived cytokines in COPD remain largely unexplored. IL-21 might act in a positive feedback loop, preserving and/or amplifying generation of Th17 cells [39, 40], and serve to recruit Th17 cells into the inflamed tissue [41]. Moreover, Leonard and Spolski [42] indicated that IL-21 could significantly increase lymphocytes survival and cytolytic potential. Given these findings, it is perhaps not surprising that exaggerated IL-17 and IL-21 responses are implicated in the pathogenesis of COPD. In the present study, significantly elevated levels of IL-17 and IL-21 were found in the peripheral blood and lungs from CS-exposed mice. In addition, a positive correlation between levels of IL-17 and IL-21 and emphysematous lesions was found in CS-exposed mice.

Although these findings suggest that the enhanced Th17 cells and IL-21 production in emphysema are biologically relevant, the mechanism for Th17 cells in emphysema pathogenesis remains unidentified. Several studies have shown that the number of CD8^+^ T cells found in the lungs of patients with COPD correlates with disease severity [43], and lung CD8^+^ T cells may directly cause cytotoxicity contributing to emphysema by inducing apoptosis through secretion of perforin, granzyme, and by Fas/Fas ligand (FasL) interactions [44]. Further, it has been demonstrated that CD4^+^ T cells are essential in the promotion of functional CD8^+^ T-cell memory after an acute infection [45]. Therefore, it is likely that Th17 cells may participate in COPD immunoregulation via generation of CD8^+^ cytotoxic T cells.

IL-21 is an effector cytokine that is made predominantly by Th17 cells [36]. It mediates its effects through a class I cytokine family receptor IL-21R, which specifically binds IL-21 [20]. In the current study, we demonstrated that the number of IL-21^+^Th17 and CD8^+^IL-21R^+^ T cells was significantly increased in the lungs and peripheral blood of CS-exposed mice. More importantly, a significantly positive correlation between the number of IL-21^+^Th17 and CD8^+^IL-21R^+^ T cells and emphysematous lesions was found in CS-exposed mice, which indicated the importance of IL-21^+^Th17 and CD8^+^IL-21R^+^ T cells in the development of emphysema. In addition, a significant correlation existed between IL-21^+^Th17 and CD8^+^IL-21R^+^ T cells in the lungs and peripheral blood of CS-exposed mice. This suggests that Th17 cells could produce IL-21 to mediate inflammatory response through IL-21R, which is expressed on CD8^+^ T cells. Thus, we presume that IL-21 released from Th17 cells might play a more important role in the immunopathology of emphysema through its actions on CD8^+^ T cells.

To further address this issue, we examined the capacity of rIL-21 to promote CD8^+^ T cells functions *in vitro* and found that coculture of IL-21 and CD8^+^ T cells results in cells producing increased amounts of perforin and granzyme-B upon chronic smoke expose. Interestingly, the CD8^+^ perforin^+^ cells were proportionally higher than CD8^+^ granzyme B^+^ cells, and the perforin levels were also much higher than the granzyme B levels. Perforin can form pores in the target cells' membranes, while granzymes, as serine proteases, enter the cytoplasm of the target cells, altering their function and/or activating cell death [46]. These observations indirectly demonstrate that IL-21 produced by Th17 cells can promote CD8^+^ T cells to induce apoptosis and tissue damage via the granzyme-B/perforin-mediated pathway. However, the precise mechanism for the effect of Th17 cells on the development of smoke-induced emphysema undoubtedly needed successive studies.

## 5. Summary

Our data showed that the frequencies of Th17, IL-21^+^Th17, and CD8^+^IL-21R^+^ T cells and the levels of IL-17 and IL-21 in the peripheral blood and lungs of CS-exposed mice were significantly increased compared to controls and correlated with emphysematous lesions. Furthermore, expression of perforin and granzyme B by CD8^+^ T cells was increased by *in vitro* stimulation with IL-21. Our findings support the concept that Th17 cells and related cytokine IL-21 were involved in the pathogenesis of COPD. However, to the best of our knowledge, although in the *in vitro* experiments IL-21 promotes CD8^+^ T cell cytotoxic responses, whether or not IL-21 derived from the Th17 cells contributes to the enhanced CD8^+^ T cells function response remains unclear. Further research should be done by using adoptive transfer of Th17 cells and IL-21-null (or IL-21 deficient) mice.

## References

[1] Global Initiative for Chronic Obstructive Lung Disease Global strategy for the diagnosis, management, and prevention of chronic obstructive pulmonary disease. http://www.goldcopd.com/.

[2] National Institute for Health and Clinical Excellence Chronic obstructive pulmonary disease: management of chronic obstructive pulmonary disease in adults in primary and secondary care. http://guidance.nice.org.uk/CG101/Guidance/pdf/English.

[3] Niewoehner DE (2010). Outpatient management of severe COPD. *The New England Journal of Medicine*.

[4] Cosio MG, Majo J, Cosio MG (2002). Inflammation of the airways and lung parenchyma in COPD: role of T cells. *Chest*.

[5] Agustí A, MacNee W, Donaldson K, Cosio M (2003). Hypothesis: does COPD have an autoimmune component?. *Thorax*.

[6] Lee SH, Goswami S, Grudo A (2007). Antielastin autoimmunity in tobacco smoking-induced emphysema. *Nature Medicine*.

[7] Rouvier E, Luciani MF, Mattei MG, Denizot F, Golstein P (1993). CTLA-8, cloned from an activated T cell, bearing AU-rich messenger RNA instability sequences, and homologous to a herpesvirus Saimiri gene. *Journal of Immunology*.

[8] Kawaguchi M, Onuchic LF, Li XD (2001). Identification of a novel cytokine, ML-1, and its expression in subjects with asthma. *Journal of Immunology*.

[9] Hizawa N, Kawaguchi M, Huang SK, Nishimura M (2006). Role of interleukin-17F in chronic inflammatory and allergic lung disease. *Clinical and Experimental Allergy*.

[10] Di Stefano A, Caramori G, Gnemmi I (2009). T helper type 17-related cytokine expression is increased in the bronchial mucosa of stable chronic obstructive pulmonary disease patients. *Clinical and Experimental Immunology*.

[11] Pelletier M, Maggi L, Micheletti A (2010). Evidence for a cross-talk between human neutrophils and Th17 cells. *Blood*.

[12] Chen Y, Thai P, Zhao YH, Ho YS, DeSouza MM, Wu R (2003). Stimulation of airway mucin gene expression by interleukin (IL)-17 through IL-6 paracrine/autocrine loop. *Journal of Biological Chemistry*.

[13] Park H, Li Z, Yang XO (2005). A distinct lineage of CD4 T cells regulates tissue inflammation by producing interleukin 17. *Nature Immunology*.

[14] Nistala K, Moncrieffe H, Newton KR, Varsani H, Hunter P, Wedderburn LR (2008). Interleukin-17-producing T cells are enriched in the joints of children with arthritis, but have a reciprocal relationship to regulatory T cell numbers. *Arthritis and Rheumatism*.

[15] Tzartos JS, Friese MA, Craner MJ (2008). Interleukin-17 production in central nervous system-infiltrating T cells and glial cells is associated with active disease in multiple sclerosis. *American Journal of Pathology*.

[16] Chang Y, Nadigel J, Boulais N (2011). CD8 positive T cells express IL-17 in patients with chronic obstructive pulmonary disease. *Respiratory Research *.

[17] Shan M, Cheng HF, Song LZ (2009). Lung myeloid dendritic cells coordinately induce TH1 and TH17 responses in human emphysema. *Science Translational Medicine*.

[18] Monteleone G, Pallone F, Macdonald TT (2009). Interleukin-21 as a new therapeutic target for immune-mediated diseases. *Trends in Pharmacological Sciences*.

[19] Nurieva R, Yang XO, Martinez G (2007). Essential autocrine regulation by IL-21 in the generation of inflammatory T cells. *Nature*.

[20] Mehta DS, Wurster AL, Grusby MJ (2004). Biology of IL-21 and the IL-21 receptor. *Immunological Reviews*.

[21] Zeng R, Spolski R, Finkelstein SE (2005). Synergy of IL-21 and IL-15 in regulating CD8^+^ T cell expansion and function. *Journal of Experimental Medicine*.

[22] D’hulst AI, Vermaelen KY, Brusselle GG, Joos GF, Pauwels RA (2005). Time course of cigarette smoke-induced pulmonary inflammation in mice. *European Respiratory Journal*.

[23] Macdonald G, Kondor N, Yousefi V, Green A, Wong F, Aquino-Parsons C (2004). Reduction of carboxyhaemoglobin levels in the venous blood of cigarette smokers following the administration of carbogen. *Radiotherapy and Oncology*.

[24] Saetta M, Shiner RJ, Angus GE (1985). Destructive index: a mesurement of lung parenchymal destruction in smokers. *American Review of Respiratory Disease*.

[25] Thurlbeck WM (1967). Measurement of pulmonary emphysema. *American Review of Respiratory Disease*.

[26] Vermaelen KY, Carro-Muino I, Lambrecht BN, Pauwels RA (2001). Specific migratory dendritic cells rapidly transport antigen from the airways to the thoracic lymph nodes. *Journal of Experimental Medicine*.

[27] Voßhenrich CAJ, Di Santo JP (2001). Cytokines: IL-21 joins the *γ*c-dependent network?. *Current Biology*.

[28] Li Y, Bleakley M, Yee C (2005). IL-21 influences the frequency, phenotype, and affinity of the antigen-specific CD8 T cell response. *Journal of Immunology*.

[29] Barnes PJ, Cosio MG (2004). Characterization of T lymphocytes in chronic obstructive pulmonary disease. *PLoS Medicine*.

[30] Reiner SL (2007). Development in motion: helper T cells at work. *Cell*.

[31] Sakaguchi S, Sakaguchi N (2005). Regulatory T cells in immunologic self-tolerance and autoimmune disease. *International Reviews of Immunology*.

[32] Steinman L (2007). A brief history of TH17, the first major revision in the T H1/TH2 hypothesis of T cell-mediated tissue damage. *Nature Medicine*.

[33] Qiu SL, Bai J, Zhong XN, Huang QP, Chen H, Liu GN (2010). CD4^+^Foxp3^+^ regulatory T cells in inflammation and emphysema after smoking cessation in rats. *Chinese Journal of Tuberculosis and Respiratory Diseases*.

[34] Duan MC, Zhong XN, He ZY, Tang HJ, Huang Y (2011). Effect of interleukin-17-producing CD4^+^ T helper lymphocytes on cigarette smoke-induced lung inflammation and emphysema in mice. *Chinese Journal of Tuberculosis and Respiratory Diseases*.

[35] Harrison OJ, Foley J, Bolognese BJ, Long E, Podolin PL, Walsh PT (2008). Airway infiltration of CD4^+^CCR6^+^ Th17 type cells associated with chronic cigarette smoke induced airspace enlargement. *Immunology Letters*.

[36] Korn T, Bettelli E, Oukka M, Kuchroo VK (2009). IL-17 and Th17 cells. *Annual Review of Immunology*.

[37] Prause O, Bozinovski S, Anderson GP, Lindén A (2004). Increased matrix metalloproteinase-9 concentration and activity after stimulation with interleukin-17 in mouse airways. *Thorax*.

[38] Alcorn JF, Crowe CR, Kolls JK (2009). TH17 cells in asthma and COPD. *Annual Review of Physiology*.

[39] Korn T, Bettelli E, Gao W (2007). IL-21 initiates an alternative pathway to induce proinflammatory T H17 cells. *Nature*.

[40] Nurieva R, Yang XO, Martinez G (2007). Essential autocrine regulation by IL-21 in the generation of inflammatory T cells. *Nature*.

[41] Monteleone G, Pallone F, MacDonald TT (2008). Interleukin-21: a critical regulator of the balance between effector and regulatory T-cell responses. *Trends in Immunology*.

[42] Leonard WJ, Spolski R (2005). Interleukin-21: a modulator of lymphoid proliferation, apoptosis and differentiation. *Nature Reviews Immunology*.

[43] O’Shaughnessy TC, Ansari TW, Barnes NC, Jeffery PK (1997). Inflammation in bronchial biopsies of subjects with chronic bronchitis: inverse relationship of CD8^+^ T lymphocytes with FEV1. *American Journal of Respiratory and Critical Care Medicine*.

[44] Chrysofakis G, Tzanakis N, Kyriakoy D (2004). Perforin expression and cytotoxic activity of sputum CD8^+^ lymphocytes in patients with COPD. *Chest*.

[45] Sun JC, Williams MA, Bevan MJ (2004). CD4^+^ T cells are required for the maintenance, not programming, of memory CD8^+^ T cells after acute infection. *Nature Immunology*.

[46] Trapani JA, Smyth MJ (2002). Functional significance of the perforin/granzyme cell death pathway. *Nature Reviews Immunology*.

